# Repetitive transcranial magnetic stimulation: advances for psychiatric disorders

**DOI:** 10.3389/fpsyt.2026.1832754

**Published:** 2026-08-19

**Authors:** Zhaoyang Wu, Yunchao Xu, Qing Huang, Bojia Liu, Shiyu Zhou

**Affiliations:** Department of Psychiatry, Dalian Medical University, Dalian, Liaoning, China

**Keywords:** clinical efficacy, major challenges, neuromodulation, psychiatric disorders, repetitive transcranial magnetic stimulation (rTMS)

## Abstract

Repetitive transcranial magnetic stimulation (rTMS) has accumulated substantial exploratory evidence in the treatment of major Depressive Disorders, bipolar disorder, schizophrenia, obsessive-compulsive disorder, and generalized anxiety disorder; however, its clinical efficacy is highly dependent on the precise matching of stimulation parameters (such as frequency, intensity, total number of pulses, and treatment course) with target regions (such as the dorsolateral prefrontal cortex, supplementary motor area, temporal-parietal cortex). Key findings: In major Depressive Disorders, both high-frequency stimulation of the left dorsolateral prefrontal cortex and low-frequency stimulation of the right dorsolateral prefrontal cortex demonstrate antidepressant effects; however, efficacy is modulated by coil type, concomitant medication, and disease subtypes, and there is currently a lack of reliable closed-loop biomarker guidance for individualized treatment. Research on bipolar disorder suggests that intermittent theta burst stimulation and low-frequency right dorsolateral prefrontal cortex stimulation are effective for depressive episodes, but evidence for manic episodes is limited, necessitating the establishment of rigorous risk management mechanisms for manic conversion. In the treatment of schizophrenia, low-frequency temporo-parietal stimulation can alleviate auditory hallucinations, while improvements in negative symptoms and cognitive deficits are associated with high frequency, high intensity, long treatment duration, and younger patients; however, there is significant heterogeneity among studies. Regarding obsessive-compulsive disorder, low-frequency stimulation of the right dorsolateral prefrontal cortex, low-frequency stimulation of the supplementary motor area, and deep transcranial magnetic stimulation targeting the orbitofrontal cortex all demonstrate therapeutic potential; however, treatment efficacy shows extremely high heterogeneity (I² > 75%), and there is an urgent need to establish uniform criteria for classifying treatment resistance. Preliminary evidence for generalized anxiety disorder supports high-frequency stimulation of the right dorsolateral prefrontal cortex and bilateral combined protocols; their efficacy may involve the regulation of brain-derived neurotrophic factor and serotonin levels, but current studies have small sample sizes and the mechanisms of action remain unclear. Although these findings support the potential application of rTMS in various psychiatric disorders, current studies generally share common limitations, including insufficient sample sizes, lack of rigorous sham controls, short follow-up periods, absence of standardized parameters, and scarcity of reliable biomarkers, which severely hinder its clinical implementation. Future research urgently requires the establishment of standardized stimulation parameters and a treatment-resistance grading framework through multicenter, large-sample randomized controlled trials. It should integrate neuro-navigation with neurophysiological biomarkers (such as resting-state functional MRI and individualized alpha frequency) to achieve personalized, precision-targeted treatment, and systematically evaluate the synergistic effects of rTMS with medication and psychotherapy, as well as its long-term safety.

## Introduction

Mental illness encompasses a spectrum of disorders marked by dysfunction of the brain, which results from complex interactions among biological, psychological, and social factors. These disorders are expressed as disturbances in cognition, emotion, and volitional behavior, severely impairing affected individuals’ psychological health and social functioning (1). Common psychiatric conditions include Depressive Disorders, bipolar disorder (BD), schizophrenia, obsessive-compulsive disorder (OCD), and anxiety disorders. All represent formidable therapeutic challenges (2). Mental disorders are the leading cause of the global burden of disability-adjusted life years (3). Despite implementing a series of effective mental disorder prevention and control measures in recent years, China remains one of the countries with the most severe mental disorder burden globally due to its large population base (4). Existing treatment algorithms for these disorders primarily feature pharmacological interventions. However, current psychotropic agents frequently cause significant adverse effects, including but not limited to seizure induction, extrapyramidal symptoms (EPS), neuroleptic malignant syndrome (NMS), and multi-organ toxicity. Additionally, a considerable subset of patients exhibits poor pharmacological responsiveness or intolerable side effects associated with these medications (5), underscoring the urgent demand for safer and more effective treatment modalities, as well as the pursuit of innovative therapeutic avenues. Against this backdrop, repetitive transcranial magnetic stimulation (rTMS)—a novel, noninvasive neuromodulation methodology—has attracted pronounced attention within the psychiatric treatment landscape in recent years.

Transcranial Magnetic Stimulation (TMS) was introduced by Anthony T. Baker in 1985. Utilizing the principle of electromagnetic induction, it non-invasively elicits compound action potentials in the hand muscles via the human motor cortex. This technique not only enables objective assessment of the corticospinal tract pathway but also marks a pivotal breakthrough in translating non-invasive brain modulation technology from theory to practice (6). Its technical foundation stems from time-varying magnetic fields inducing secondary currents within neural tissue, thereby causing neuronal depolarization and triggering a cascade of neural responses (7). With the development of repetitive application protocols (i.e., rTMS), sustained changes in synaptic plasticity, specifically long-term potentiation (LTP) or long-term depression (LTD) effects (8). It is generally accepted that high-frequency repetitive transcranial magnetic stimulation (HF-rTMS, ≥5 Hz) enhances cortical excitability, whereas low-frequency repetitive transcranial magnetic stimulation (LF-rTMS, ≤1 Hz) tends to produce inhibitory effects (9, 10). However, this “high-frequency excitation/low-frequency inhibition” model lacks universality. Its actual effects are modulated by multiple factors including individual pathological states, target neural network architecture, and stimulation parameters, exhibiting significant context dependency.

As a safe, non-invasive neuromodulation therapy, rTMS has received approval from the U.S. Food and Drug Administration (FDA) for clinical use in Major Depressive Disorders (MDD) (11). Converging evidence from clinical observations, neuroimaging modalities (including functional magnetic resonance imaging and positron emission tomography), and neuroanatomical research reveal substantial dysfunctions within the prefrontal cortex—particularly the dorsolateral prefrontal cortex (DLPFC)—and limbic regions (such as the amygdala and anterior cingulate cortex[ACC]) among individuals with Depressive Disorders. These brain regions are integral to emotional regulation processes (12). rTMS induces neuronal depolarization and activates long-term neuroplasticity mechanisms by applying time-varying magnetic fields to specific brain regions (e.g., the left DLPFC) (8), thereby potentially modulating neural circuits involved in emotional processing (13). It is worth emphasizing that existing evidence is largely based on correlational neuroimaging studies. The regulatory mechanism of rTMS on emotional circuits may involve modulating the dynamic equilibrium between the default mode network and the salience network, rather than a simple unidirectional causal relationship of excitatory and inhibitory mechanisms.

To further enhance therapeutic efficiency, intermittent theta burst stimulation (iTBS) has been developed as a novel rTMS technique. By mimicking cortical theta rhythms, it enhances synaptic transmission and cortical excitability. The iTBS protocol substantially reduces single-session duration from approximately 37 minutes for conventional 10 Hz rTMS to just 3 minutes, while delivering equivalent antidepressant effects, demonstrating advantages in both time and pulse count (14). However, this time-efficient benefit comes with a significant difference in total stimulation dose: the standard 10 Hz rTMS protocol typically delivers 3,000 pulses, whereas the standard iTBS protocol administers only 600–1,200 pulses. Differences in total stimulation dose, spatiotemporal distribution, and bioequivalence between the two protocols may lead to distinct patterns of neuroplasticity responses and variations in individual treatment efficacy. Accelerated intermittent theta burst stimulation (aiTBS)—involving multiple short bursts of high-frequency stimulation per day (e.g., 4–10 daily sessions delivering several times the total pulses of conventional protocols)—may more efficiently induce neuroplastic changes. To date, TBS protocols demonstrate comparable safety and efficacy to conventional rTMS while exhibiting advantages in tolerability, therapeutic flexibility, and cost-effectiveness (14, 15).

Mechanistically, compelling studies have demonstrated that rTMS can modulate functional connectivity (FC) between distinct brain regions. For example, rTMS has been shown to enhance FC between the right temporoparietal cortex (TPC) and the right insula, suggesting that its therapeutic action may entail normalization of aberrant functional connectivity (16). Hasan et al. found in a study involving approximately 30 patients that 10 Hz left DLPFC rTMS treatment was associated with increased volume in the left hippocampus and parahippocampal gyrus, with this change showing statistically association with symptom improvement (17). It should be noted that such findings originate from exploratory analyses with small sample sizes; causal relationships remain unestablished, and the predictive value of structural changes for clinical efficacy requires validation through large-scale studies.

**Figure 1:**
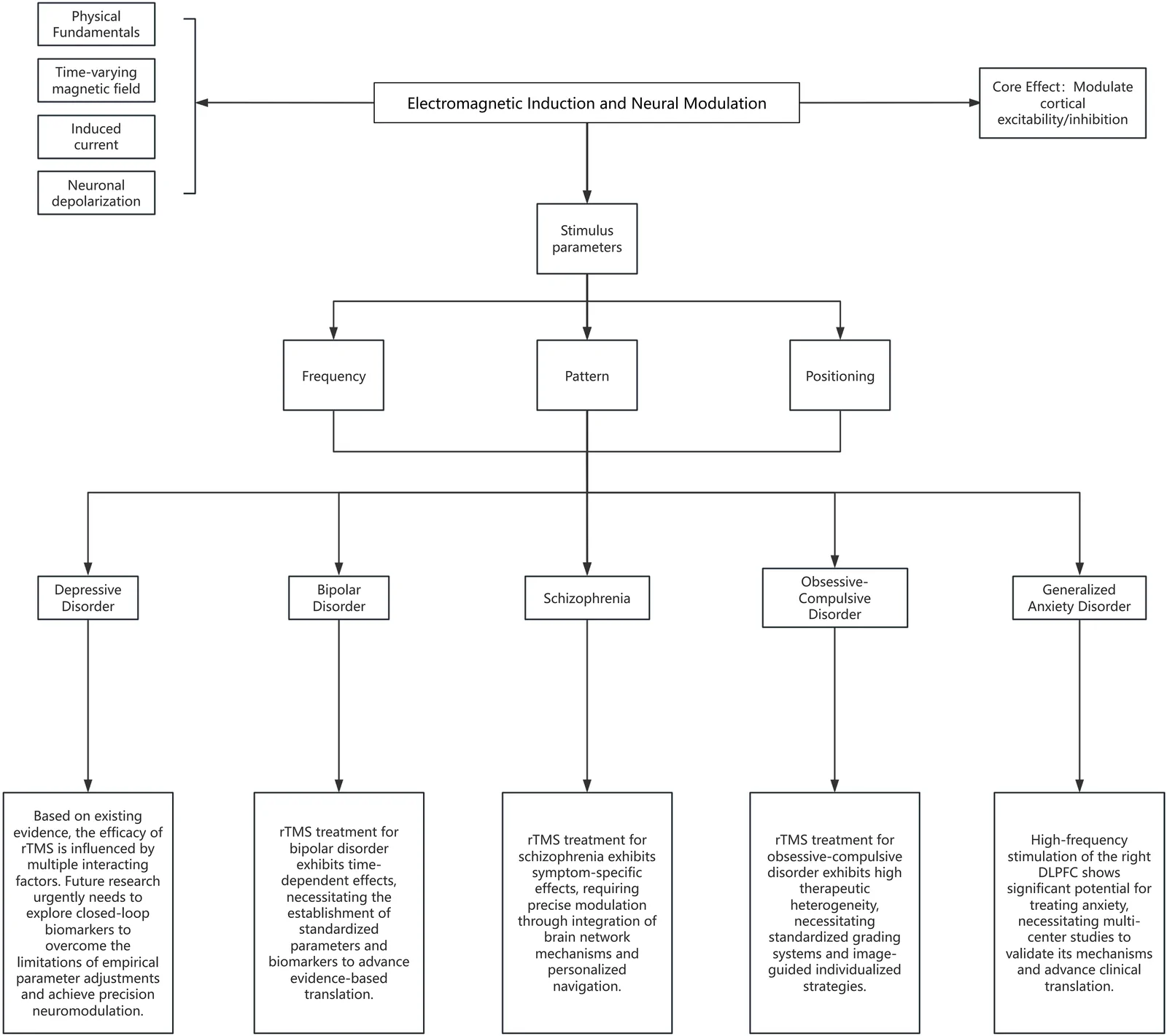
The schematic, "Graphical abstract" delineates a robust translational neuroscience framework for implementing rTMS in psychiatry. The top section details the cascading physical fundamentals: a time-varying magnetic field creates an induced current, thereby modulating cortical excitability. This foundational process informs three pivotal stimulus parameters: frequency, pattern, and positioning. These critical variables are then integrated and tailored for five major clinical conditions: Depressive Disorder, Bipolar Disorder, Schizophrenia, OCD, and Generalized Anxiety Disorder. Detailed textual summaries for each condition prioritize future research directions. Key emerging needs highlighted include the discovery of closed-loop biomarkers, the establishment of standardized parameters, and the validation of image-guided personalized strategies. This structured approach aims to transition from current empirical trial-and-error adjustments to targeted, mechanism-based precision neuromodulation across various psychiatric pathologies.

Beyond Depressive Disorders, rTMS has been investigated for conditions such as schizophrenia, bipolar disorder, and generalized anxiety disorder (GAD) (**Figure 1**). Regarding schizophrenia, its positive symptoms correlate with dysfunction in the frontotemporal circuitry; applying prefrontal rTMS may alleviate symptoms through a trans-synaptic regulatory mechanism (18). In OCD, rTMS research has primarily targeted functional regulation of the orbitofrontal-striatal-thalamic neurocircuitry, engaging sites such as the DLPFC, ACC, supplementary motor area (SMA), orbital frontal cortex (OFC), and medial prefrontal cortex (mPFC) (19–21). Abnormalities in the recursive connectivity of the Cortico-Striato-Thalamo-Cortical Loop (CSTC) are closely associated with OCD pathogenesis, though therapeutic parameters for rTMS in this domain remain unstandardized (22). For GAD, neuroimaging findings implicate functional disturbances in the frontal-limbic system and dorsomedial prefrontal cortex (23). and right DLPFC-targeted rTMS has demonstrated efficacy in alleviating GAD symptoms by modulating this region’s neural activity (24). Additionally, many GAD patients show hyperconnectivity between the limbic and prefrontal cortices, suggesting a plausible mechanism whereby rTMS restores functional network balance (25). However, these findings are largely derived from small-sample studies, and their clinical translational value requires further confirmation.

In summary, rTMS demonstrates significant clinical value and potential in treating psychiatric disorders. To systematically review advances in this field, this paper employs a systematic literature review methodology, with the specific research approach outlined below. Databases searched included PubMed, Embase, Web of Science, and China National Knowledge Infrastructure (CNKI). Search keywords were combined around the following themes: Mental Disorders, Psychiatric Disorders, rTMS, and specific diagnoses such as Depressive Disorders, Bipolar Disorder, Schizophrenia, Obsessive-Compulsive Disorder, Anxiety Disorder, and related efficacy terms (e.g., response rate, efficacy). The search period spanned from each database’s inception to December 31, 2024. Included studies primarily comprised original research, randomized controlled trials, and systematic reviews published in Chinese or English, excluding case reports, conference abstracts, and studies on unrelated diseases. We conducted a comprehensive literature search using a systematic search strategy. On this basis, we performed a narrative synthesis of high−quality studies (primarily randomized controlled trials and meta−analyses), while noting the methodological limitations of individual studies where appropriate (**Table 1**).

**Table 1:** Comparative schematic of rTMS for the treatment of psychiatric disorders.

Disease types	Stimulation protocol/target	Representative evidence (sample size, design, effect)	Evidence level	Major challenges
Depressive Disorders	Left DLPFC high−frequency (HF−rTMS); bilateral rTMS	Multiple meta−analyses confirm efficacy of HF−rTMS and LF−rTMS, but most original studies have poor blinding or inadequate sham control, potentially overestimating effects 2024 meta−analysis: 1200−1500 pulses/session associated with better response, but moderated by coil type and disease subtype Bilateral vs left−sided: systematic review of 7 RCTs showed no significant difference in most studies; only 3 found active bilateral > sham.	Established (efficacy exists, but effect size may be inflated)	Lack of reliable biomarkers; parameter interactions unclear; insufficient research on combination with pharmacotherapy
Bipolar Disorder (depressive phase)	aiTBS/low−frequency right DLPFC; dTMS (H1−coil)	Small RCT: aiTBS improves treatment−resistant bipolar depression; hippocampal volume increased (mechanism unclear, not a validated biomarker). Low−frequency (1 Hz) right DLPFC effective; unilateral and bilateral sequential protocols show similar efficacy (small samples). dTMS shows acute efficacy (single−center study).	Exploratory (small samples, no confirmatory trials)	High−frequency stimulation requires monitoring for manic switch; small sample sizes, large variability in sham methods
Bipolar Disorder (manic phase)	High−frequency (10−20 Hz) right DLPFC	Adult small RCT (n=27, 2 weeks): active > sham; adolescent RCT: negative; early RCT suggested possible efficacy.	Conflicting/limited (no consistent conclusion)	Very limited evidence; not recommended for clinical use
Schizophrenia (cognition/negative symptoms)	High−frequency (10−20 Hz) prefrontal cortex	20 Hz bilateral (4 weeks): improved working memory (small RCT); 10 Hz left DLPFC large trial (n=156, 3 weeks): not superior to sham. Negative symptoms: meta−analysis shows efficacy (effect size 0.49−0.64), associated with high frequency, longer course, younger age.	Conflicting (cognitive efficacy highly parameter−dependent)	High heterogeneity in cognitive results; small effect size, uncertain clinical significance
Schizophrenia (auditory hallucinations)	Low−frequency (1 Hz) temporoparietal cortex	Multiple RCTs confirm efficacy; but response depends on localisation accuracy (MRI−guided > 10−20 system) and stimulation intensity/course.	Established (efficacy exists, but with high heterogeneity)	Non−uniform localisation methods; large differences in sham control
Obsessive−Compulsive Disorder	Low−frequency SMA; low−frequency right DLPFC; dTMS (OFC/mPFC)	Meta−analysis: overall superior to sham (Hedge’s g 0.45−0.79), but SMA studies have extremely high heterogeneity (I² > 75%). Network meta−analysis (methodological limitations) suggests right DLPFC low−frequency or bilateral DLPFC high−frequency as preferred (SUCRA > 80%). dTMS + behavioural therapy: small−study potential in refractory OCD.	Conflicting (overall effective but very high heterogeneity; optimal protocol uncertain)	No standard definition of treatment resistance; lack of standardised parameters may inflate efficacy estimates
Generalized Anxiety Disorder	High−frequency (20 Hz) right DLPFC	Small study (n=40, 4 weeks): remission >70%, η² = 0.91, but small sample and short duration may overestimate effect. Bilateral low−frequency (n=28): anxiety reduction correlated with serum BDNF/5−HT increase (correlational, not causal).	Exploratory (no large RCT validation)	Evidence from small, short−term, open−label studies; neural mechanisms still hypothetical

Note on evidence levels: “Established” may still include inflation or heterogeneity; “Conflicting/High heterogeneity” indicates inconsistent results; “Exploratory” refers to small−sample or unreplicated findings. All quantitative claims are linked to specific studies or meta−analyses.

## Depressive disorders

Depressive Disorders is a prevalent, chronic psychiatric illness whose core symptoms include sustained and significant low mood, marked reduction in interest, and loss of pleasure (anhedonia). In severe cases, patients may present with psychotic features—such as hallucinations or delusions—and face considerable behavioral risks, particularly self-harming behavior and suicide (26). Standard therapeutic options comprise pharmacological interventions—including selective serotonin reuptake inhibitors, serotonin-norepinephrine reuptake inhibitors, norepinephrine-dopamine reuptake inhibitors, tricyclic antidepressants, and monoamine oxidase inhibitors—as well as non-pharmacological techniques such as physical therapies and psychotherapy (27). Despite this diversity of evidence-based treatments, a notable proportion of patients experience incomplete remission or difficulty in sustaining recovery, underscoring the necessity to develop novel interventions and optimize multimodal therapeutic strategies to improve outcomes in Depressive Disorders.

Patients with MDD receiving twice-daily HF-rTMS treatment achieved clinical response and remission rates of 55.6% and 37%, respectively, after two weeks (28). It should be noted that this study has the following limitations: the treatment period was relatively short (which could be extended if efficacy improves accordingly), follow-up time was limited, the sample size was small (n=27), and an open-label design was used, necessitating validation through randomized controlled trials. Therefore, these findings should be regarded as exploratory evidence with limited clinical generalizability. Another randomized controlled trials demonstrated that HF-rTMS accelerates and enhances clinical response to antidepressants in major Depressive Disorders (29). However, most of these studies suffered from inadequate blinding or insufficient control of sham stimulation, and potentially overestimated the actual effects. Previous research has confirmed that both HF-rTMS and LF-rTMS possess certain antidepressant efficacy (30, 31). Meta-analyses suggest that HF-rTMS may demonstrate superior efficacy compared to LF-rTMS (31), although LF-rTMS exhibits better patient tolerability than HF-rTMS (32). Notably, a 2024 meta-analysis indicated that increasing both the number of stimulation sessions and the total pulses per session (with an optimal range of 1200 to 1500 pulses per session) is significantly associated with enhanced antidepressant effects of left DLPFC HF-rTMS (33). However, the total pulse count itself is not an independent predictor, as its effect is modulated by factors such as coil type, target depth, and disease subtype. For instance, the distinct electric field distribution characteristics of circular versus figure-of-eight coils may influence stimulation precision and depth penetration. Patients with Depressive Disorders exhibiting psychotic features may require higher stimulation intensities. Crucially, these critical variables remain under-explored in existing research, and the interplay between parameters continues to present both a primary focus and a significant challenge for current investigations.

Regarding the selection of stimulation protocols, discrepancies exist in some research findings. A systematic review and meta-analysis evaluated seven randomized controlled trials on HF-rTMS for major Depressive Disorders. Results indicated that bilateral DLPFC repetitive transcranial magnetic stimulation (right LF-rTMS and left HF-rTMS) showed no significant difference in efficacy compared to unilateral left HF-rTMS in most studies. Only one study suggested superior efficacy for bilateral stimulation, while two studies reported lower efficacy for bilateral stimulation. Furthermore, when comparing bilateral rTMS to sham stimulation, only three studies demonstrated a significant therapeutic advantage for active stimulation over sham (34). This inconsistency may stem from multiple factors: Firstly, it remains uncertain whether standard 1 Hz right DLPFC stimulation effectively inhibits the prefrontal-limbic circuity in all individuals; fMRI studies indicate significant variability in the neural activity patterns induced by identical parameters across different subjects. Secondly, heterogeneity exists across studies regarding patient baseline characteristics, stimulation parameters, and efficacy assessment, necessitating cautious interpretation of direct comparative results.

Studies examining combined antidepressant pharmacotherapy indicate that most patients receiving rTMS concurrently utilize antidepressant medications (35). Notably, factors such as drug type, treatment duration, and plasma drug concentrations may modulate rTMS response, and the efficacy of combined therapy may vary across patient populations; however, current research in this area remains insufficient. Some studies indicate that in patients with mild to moderate Depressive Disorders, the combination of rTMS and antidepressants shows no statistically significant difference compared to antidepressant monotherapy (36). Furthermore, both approaches demonstrate comparable acceptability (measured by discontinuation rates) and tolerability (measured by discontinuation rates due to adverse events), consistent with previous findings (29, 37). However, in treatment-resistant depression (TRD) patients, particularly those requiring urgent intervention due to factors such as suicidal risk, HF-rTMS combined with antidepressants may demonstrate more pronounced clinical advantages. Notably, while both rTMS and ketamine alone exhibit significant antidepressant effects, some patients fail to achieve expected improvement. Recent studies suggest that combining the two may produce synergistic effects through distinct mechanisms, potentially yielding stronger and more sustained therapeutic outcomes (38). However, particular attention should be paid to safety concerns associated with combined use, including increased neuroexcitatory load, risk of seizures, and acute psychotic fluctuations. Future research should expand sample sizes, adopt randomized controlled designs, and establish standardized combined treatment protocols to thoroughly investigate mechanisms of action and safety profiles.

Beyond the aforementioned intervention approaches, the integrated model combining rTMS with psychotherapy also demonstrates promising applications. Real-world studies support the clinical efficacy of rTMS, and integrating psychological interventions may further enhance outcomes (39).

Although preliminary research findings align with clinical expectations, there remains a lack of reliable and broadly applicable non-invasive neurophysiological biomarkers—brain signals that stably reflect Depressive Disorders states and correlate significantly with clinical outcomes—to guide rTMS treatment (40). Such biomarkers typically operate on the principle of closed-loop systems, dynamically adjusting stimulation parameters through real-time neural feedback to achieve precise control over neurophysiological changes. An ideal closed-loop biomarker must satisfy two stringent criteria: firstly, cross-individual reproducibility, demonstrating stable presentation across a broad population of depressed individuals rather than being confined to specific samples; secondly, a strong and significant correlation with Depressive Disorders to ensure clinical relevance.

## Bipolar disorder

Bipolar disorder (BD) is a chronic, recurrent psychiatric condition characterized by alternating episodes of mania, hypomania, or mixed states and periods of residual mood symptoms between episodes (41). Despite the use of pharmacotherapeutics and psychosocial interventions to achieve symptomatic remission, a substantial proportion of patients continues to experience functional impairment and reduced quality of life, hindering return to pre-morbid functioning (42). A randomized controlled trial conducted in 2000 on daily left prefrontal cortical TMS (43) provided preliminary support for the application of TMS in BD populations.

Emerging clinical data indicate that accelerated intermittent theta burst stimulation (aiTBS) applied to the DLPFC produces significant improvements in depressive symptomatology in individuals with treatment-resistant BD (44). Imaging analysis revealed that compared with the sham stimulation group, the true stimulation group showed an increasing trend in ipsilateral hippocampal volume (45). It is noteworthy that significant variations exist in hippocampal volume measurement methodologies—whole-hippocampal assessments may obscure alterations in specific subregions (e.g., dentate gyrus), while observed volumetric increases could stem from diverse biological mechanisms including glial proliferation, angiogenesis, or tissue water content changes, and their clinical significance warrants cautious interpretation. Previous iTBS trials in BD patients also reported hippocampal volume increases (46), though these findings remain exploratory evidence and have not yet been established as reliable biomarkers. Furthermore, low-frequency (1 Hz) right DLPFC stimulation demonstrated efficacy (47). Different stimulation protocols (unilateral LF/HF and bilateral sequential stimulation) demonstrated comparable efficacy (48), suggesting therapeutic flexibility. However, treatment parameters (e.g., altering frequency or target site) may need to be adjusted for individual non-responders. Emerging techniques such as deep transcranial magnetic stimulation (dTMS, H1 coil), demonstrate acute efficacy in treating-resistant bipolar depression by expanding stimulation depth and range (49), though long-term maintenance effects require further optimization. MRI-guided iTBS enhances target localization precision, with long-term follow-up indicating acute responders may sustain benefits for up to one year (50).

Research on rTMS during manic episodes of bipolar disorder remains relatively scarce and inconclusive. Existing studies have focused on high-frequency (10–20 Hz) stimulation of the right dorsolateral prefrontal cortex (DLPFC). An early randomized controlled trial suggested this approach may improve manic symptoms (51). However, subsequent randomized controlled trials yielded divergent outcomes: one adult study found high-frequency rTMS significantly superior to sham stimulation (52), though it should be noted this study had a limited sample size (n=27) and a short treatment duration (2 weeks). In contrast, adolescent studies showed no significant difference, potentially related to variations in the maturity of frontal lobe neural circuits across different developmental stages (53). Furthermore, left DLPFC stimulation may interfere with the effectiveness of antimanic pharmacotherapy, underscoring the importance of accurate target selection (54). Overall, current evidence supporting rTMS during manic episodes remains limited. Patients with bipolar depression undergoing high-frequency stimulation require robust manic monitoring protocols alongside adequate mood stabilizer therapy. Key risk factors include: personal or familial history of rapid cycling, current mixed episode characteristics, history of central nervous system stimulant use, and neuroimaging evidence of limbic system hyperactivity. Specific risk management measures should encompass: pre-treatment assessment of manic severity using standardized tools such as the Young Manic Rating Scale (YMRS) to exclude high-risk individuals; initiation of treatment with low-dose trial stimulation protocols; regular monitoring of mood state changes during treatment, with standardized scales recommended at least twice weekly; and establishment of clear treatment discontinuation criteria and contingency plans.

Research on rTMS for bipolar disorder faces multiple methodological challenges, primarily including insufficient sample sizes, significant methodological variations in sham stimulation protocols, and inadequate short-term follow-up. Future studies should prioritize establishing standardized key parameters through large-scale, multicenter randomized controlled trials, encompassing stimulation frequency, treatment duration, and target sites. This should be combined with neuroimaging or neurophysiological biomarkers (e.g., beta rhythm activity) to guide precision treatment. Concurrently, synergistic effects between rTMS with medications like quetiapine require validation (55), while optimization strategies and long-term maintenance protocols for emerging techniques (e.g., iTBS, dTMS) must be refined to enhance treatment efficacy in bipolar disorder. Overall, rTMS demonstrates potential in bipolar disorder, though its clinical implementation necessitates a more robust research foundation (56).

## Schizophrenia

Schizophrenia is a complex mental disorder characterized by negative symptoms (such as a lack of motivation and emotion), positive symptoms (such as hallucinations and delusions), and persistent cognitive impairments (57). Its clinical profile encompasses positive symptoms—including delusions, disorganized speech and behavior, auditory hallucinations (AH), and other perceptual anomalies—principally arising from aberrant temporoparietal cortex (TPC) circuitry (58). Negative symptomatology is characterized by reduced emotional expression and social engagement. Severe cognitive impairments—affecting domains such as working memory, episodic and verbal memory, processing speed, and social cognition—are strongly associated with dysfunction of the DLPFC (59).

Central to schizophrenia are enduring and pervasive cognitive deficits across both social and nonsocial domains (60). Notably, these deficits persist even during periods of symptomatic remission (61) and serve as critical determinants of real-world psychosocial functioning and quality of life (62). Given the prefrontal cortex’s pivotal role in cognitive impairment (59) and its accessibility as a therapeutic target, numerous studies have applied HF- rTMS to the left or bilateral prefrontal cortex. However, results across different studies exhibit marked discrepancies, closely associated with research design, stimulation parameters, and patient characteristics. For instance, left DLPFC 10Hz rTMS treatment (over four weeks) improved cognitive abilities, particularly immediate memory, in schizophrenia patients experiencing auditory hallucinations (63). A four-week randomized controlled trial found that bilateral 20 Hz rTMS treatment to the prefrontal cortex significantly improved working memory in schizophrenia patients, demonstrating statistical superiority over the sham stimulation group (64). A recent small-sample study indicated that following two weeks of bilateral 20 Hz high-frequency rTMS treatment to the frontal lobes, patients exhibited a significant overall improvement in neurocognitive function, with efficacy persisting until the conclusion of follow-up. Further analysis revealed a positive correlation between baseline left frontal cortical thickness and treatment response (65). However, a multicenter large-sample study involving 156 patients showed that three weeks of 10 Hz left prefrontal HF-rTMS treatment did not yield significantly superior efficacy compared to the sham stimulation group (66). Another study employing a similar stimulation protocol (two-week treatment course) revealed significant improvements in social cognitive abilities, particularly in facial emotion recognition (67). These conflicting results may stem from multiple factors: studies reporting positive outcomes predominantly employed higher frequencies (20 Hz) and bilateral stimulation, whereas those yielding negative results utilized relatively conservative 10 Hz unilateral stimulation; regarding patient characteristics, significant variations existed in inclusion criteria across studies (e.g., disease duration, symptom severity, and concomitant medication); Furthermore, variations in follow-up duration (ranging from immediate assessment to four weeks) and control methodologies (such as the orientation of sham stimulation coils and auditory simulation) may also influence outcome evaluation.

Auditory hallucinations, a hallmark symptom of schizophrenia, manifest in approximately 70%-80% of patients at onset (68). rTMS treatment targeting auditory hallucinations was initially developed based on *a priori* symptom identification, as excessive activation in the speech processing regions of both temporal lobes persists during hallucinations (69), lLF- rTMS (1 Hz) intervention was employed to suppress this hyperactivity, yielding therapeutic effects (70). Recent studies indicate that increasing stimulation duration (20 min versus 10–15 minutes) and extending treatment cycles (15 sessions versus 5–10 sessions) both enhance improvements in auditory hallucinations, highlighting the importance of optimizing treatment dosage (71, 72). However, inconsistencies exist between studies, potentially attributable to variations in stimulation localization methods (structural MRI-guided versus standard 10–20 system placement), stimulation intensity (80–110% RMT), and assessment tool selection. Notably, studies reporting positive outcomes frequently employed more precise neuro-navigational localizations and higher stimulation intensities, whereas negative results may have been constrained by suboptimal localization accuracy or insufficient dosage.

At the mechanistic level, the therapeutic effects of rTMS on schizophrenia may be mediated through modulation of large-scale brain networks. Research indicates that reduced metabolic activity in the prefrontal cortex (frontal attenuation) correlates with increased electrophysiological δ-band activity originating in the right prefrontal cortex, which is associated with enhanced facial emotion recognition ability. A randomized controlled trial demonstrated that following 10Hz rTMS treatment to the left DLPFC, patients exhibited a significant reduction in resting-state electroencephalogram (EEG) delta band activity (73). It should be noted that considerable variation exists between laboratories regarding EEG equipment, electrode placement protocols, and signal preprocessing methods, posing challenges for direct inter-study comparisons. Particularly concerning studies on individualized alpha-frequency rTMS, preliminary findings indicate that treatment with rTMS at the individualized alpha frequency (8–13 Hz) – specifically at the IAF (individual alpha frequency) plus 1 Hz – enhances therapeutic efficacy and increases the degree of alpha desynchronization. This suggests a close association between alpha oscillatory neuronal activity and cognitive performance (74, 75). However, the findings also underscore the resonant characteristics of rTMS and the potential necessity of tailoring stimulation based on individualized frequencies and localizations. Clinical EEG applications continue to face technical limitations such as motor artefacts and electromyographic interference, and their practical utility in providing real-time guidance for rTMS treatment remains to be further validated.

From a neural network perspective, rTMS may exert remote regulatory effects by stimulating nodal brain regions. For instance, DLPFC stimulation not only influences local neural activity but may also affect working memory function via the frontoparietal network, whilst simultaneously modulating the dynamic equilibrium between the default mode network and salience network. These network-level alterations may indirectly contribute to improvements in negative symptoms and cognitive deficits, offering fresh insights into the therapeutic mechanisms of rTMS. Recent meta-analyses indicate that rTMS significantly ameliorates negative symptoms compared to sham stimulation, with effect sizes ranging from 0.49 to 0.64. Higher efficacy correlates with: - High-frequency stimulation (10–20 Hz) - Stronger stimulation intensity (100–110% resting motor threshold, RMT) - Longer treatment duration (>3 weeks) - Younger patients (<39 years) - Shorter disease duration (<13 years) (76, 77).

In the context of precision medicine for schizophrenia, optimizing rTMS efficacy hinges on precise anatomical localization and individualized parameter selection. Identification of specific cortical or neural circuit targets may substantially improve therapeutic outcomes. Integrating neuroimaging with noninvasive brain stimulation presents considerable promise for advancing targeted interventions (78, 79). Given the well-recognized clinical and neurobiological heterogeneity among schizophrenia patients, the implementation of adaptive, patient-specific rTMS protocols is imperative. For instance, fMRI-based assessments of scene recognition can selectively activate the anterior cingulate cortex (ACC), permitting quantification of its functional state within episodic memory circuits. This enables investigation of regulatory effects of ACC-targeted rTMS on memory networks in early psychosis, providing a framework for individualized clinical interventions (80).

## Obsessive-compulsive disorder

Obsessive-compulsive disorder (OCD) is a chronic and debilitating psychiatric disorder with a global prevalence estimated at approximately 2–3%. Core clinical features include intrusive, recurring obsessive thoughts and/or compulsive rituals or behaviors (81). Current gold-standard approaches emphasize first-line serotonin reuptake inhibitors (SSRIs) in combination with cognitive behavioral therapy (CBT) and exposure and response prevention (ERP) (82). However, around 40–60% of patients fail to achieve adequate symptom resolution with these interventions alone (83), emphasizing the pressing need for novel, evidence-based therapies for refractory OCD.

As a noninvasive neuromodulation intervention, rTMS has seen expanding clinical investigation for OCD patients unresponsive to standard SSRIs and CBT/ERP protocols. Initial studies showed that specific rTMS frequencies produce distinct cortical effects (9). Treatment for OCD should adjust stimulation parameters and targets based on restoring cortical network balance, while fully accounting for patient individuality.

Current research primarily focuses on three key targets: the dorsolateral prefrontal cortex (DLPFC), the supplementary motor area (SMA), and the orbitofrontal/medial prefrontal cortex (OFC/mPFC). The DLPFC, as a core node for cognitive control, typically employs high-frequency stimulation (10–20 Hz) to enhance its top-down inhibitory function. The SMA, serving as a hub for motor planning and execution, predominantly utilizes low-frequency stimulation (1 Hz) to modulate sensorimotor integration. The OFC/mPFC, being a key region for emotional and reward processing, is targeted via deep brain stimulation coils. It must be emphasized that significant variations across studies in patient inclusion criteria (e.g., refractory definition), treatment resistance levels (number of SSRI treatment failures), and follow-up durations (ranging from 4 to 24 weeks) directly impact the objective assessment of efficacy.

Clinical evidence indicates that the efficacy of rTMS is highly dependent on the selection of stimulation target sites and parameters. Early studies on rTMS targeting the DLPFC demonstrated significant heterogeneity in treatment outcomes, potentially linked to stimulation side (left/right) and frequency selection.; Conversely, LF-rTMS targeting the SMA showed symptomatic improvement in some randomized controlled trials (84), However, multiple meta-analyses reported extremely high heterogeneity in its efficacy (I² > 75%, indicating over 75% of effect size variation stemmed from genuine inter-study differences rather than sampling error). Such substantial heterogeneity limits the reliability and clinical generalizability of findings, necessitating extreme caution when interpreting overall effect sizes. Future research is urged to further investigate sources of heterogeneity and refine study designs-through standardizing stimulation parameters and normalizing patient inclusion criteria-to yield more robust conclusions (85). Recent network meta-analyses (NMAs) indicated that left DLPFC LF-rTMS and bilateral DLPFC HF-rTMS may represent optimal treatment strategies (cumulative ranking curve area under the curve [SUCRA] value > 80%). However, it must be noted that network meta-analyses possess methodological limitations: the number of available studies is limited and unevenly distributed across target-parameter combinations; the absence of multicenter randomized controlled trials increases uncertainty in indirect comparisons; and SUCRA values reflect only relative ranking probabilities and cannot alone serve as confirmatory evidence of efficacy. Furthermore, dTMS targeting the OFC/mPFC combined with behavioral therapy demonstrates significant therapeutic potential for treatment-resistant patients (86, 87). Research on theta burst stimulation (e.g., iTBS) remains relatively limited, with only a few randomized controlled trials indicate that iTBS applied to the anterior cingulate cortex significantly reduces Yale-Brown Obsessive Compulsive Scale (Y-BOCS) scores (88). Its long-term efficacy and specific mechanisms require further investigation in large-scale studies. Despite meta-analyses indicating overall superior efficacy of rTMS over sham stimulation (Hedge’s g=0.45–0.79), significant controversy persists within the field: The absence of a unified definition of treatment resistance (e.g., a Y-BOCS reduction threshold <25% for SSRI-resistant cases) leads to conflicting subgroup analysis conclusions-some studies even suggest rTMS is more effective for non-resistant individuals (89). Concurrently, the absence of clear standards regarding stimulation site localization (left/right DLPFC), differing treatment course durations (10–30 sessions), and control group design (sham stimulation localization bias) may inflate efficacy estimates (90).

Future research in OCD should prioritize three core domains: Firstly, establishing a standardized grading framework for treatment resistance (integrating SSRI duration, dosage, and Y-BOCS trajectories) to facilitate precise stratification of likely responders; Secondly, leveraging advanced targeting capabilities through resting-state functional MRI (fMRI) or diffusion tensor imaging (DTI) to dissect abnormal network connections (e.g., OFC-thalamic hyperactivation); Finally, conducting multicenter randomized controlled trials to directly compare long-term safety and efficacy profiles of dTMS and emerging neuromodulation protocols (e.g., iTBS), thereby producing robust, translatable evidence for clinical deployment.

## Generalized anxiety disorder

Anxiety disorders—encompassing generalized anxiety disorder (GAD), panic disorder, social anxiety disorder, agoraphobia, and specific phobias—affect up to 33.7% of the population over the lifetime (91). Among these, GAD is the most prevalent, defined by chronic, relapsing symptoms of pathological anxiety and excessive, uncontrollable worry (92). Standard therapeutic interventions for GAD comprise pharmacotherapy (primarily SSRIs and serotonin-norepinephrine reuptake inhibitors) and psychotherapy, such as CBT (93). However, roughly 50% of GAD patients exhibit poor response to conventional treatments, and systematic research into mechanisms of treatment resistance remains sparse (94). There is a distinct lack of robust evidence regarding second-line or adjunctive interventions for GAD, reinforcing the critical necessity for brain-based targeted treatment development (95). rTMS has been shown to attenuate neuroinflammation and decrease the release of inflammatory mediators—mechanisms believed to underlie its ability to ameliorate mood disturbance (96). rTMS efficacy is considered comparable to other standard interventions for anxiety disorders (97), and it may yield additional clinical benefit in patients presenting with suicidality (98).

Clinical studies indicate that stimulating the right DLPFC at a frequency of 20 Hz and 110% of the resting motor threshold (RMT) yields optimal therapeutic efficacy (effect size η² = 0.91, indicating this stimulation protocol accounts for 91% of therapeutic variance, reflecting a pronounced localized effect). The remission rate exceeds 70%, demonstrating a marked superiority over conventional low-frequency protocols (99). However, while the statistical metrics underpinning these findings suggest significant effects, they may overestimate the true effect in the context of a small sample (n=40) and short treatment period (4 weeks). Consequently, the overall conclusions require validation through larger-scale, longer-follow-up replication studies. Future research should also better control for potential confounding variables to enhance the generalizability of the results. An exploratory study involving 28 participants reported that following bilateral LF-rTMS over the dorsolateral prefrontal cortex, patients exhibited significantly reduced HARS scores alongside marked increases in serum brain-derived neurotrophic factor (BDNF) and 5-hydroxytryptamine (5-HT). Serum BDNF was measured using enzyme-linked immunosorbent assay (ELISA), while 5-HT levels were determined by high-performance liquid chromatography. Pearson correlation analysis revealed a positive correlation between elevated serum 5-HT levels and BDNF levels, and a negative correlation between reduced anxiety scores and changes in BDNF and 5-HT levels. This suggests bilateral LF-rTMS may alleviate GAD by modulating the mechanisms regulating BDNF and 5-HT release within the brain (100). However, these findings originate from single-center, small-sample study. Further validation is required regarding the stability of the detection methods, inter-individual variability, and the consistency between these peripheral indicators and actual central nervous changes. Presently, the majority of evidence concerning neurochemical mechanisms derives from animal models or exploratory studies with limited sample sizes, presenting significant limitations in their extrapolation to clinical practice.

For patients with comorbid Depressive Disorders, a bilateral combined stimulation protocol is recommended: applying 10 Hz high-frequency excitatory stimulation to the left DLPFC and 1 Hz low-frequency inhibitory stimulation to the right DLPFC, achieving an 84.6% remission rate for GAD and a 76.9% remission rate for depression (101). Recent studies further indicate that paired associative stimulation (PAS) at bilateral frontal-parietal sites (rd-ccPAS-1500) proves more effective in alleviating anxiety symptoms than single-point stimulation (e.g., right dorsolateral prefrontal cortex or right posterior parietal cortex, each receiving 1500 pulses). This approach also suggests that pulse frequency may further enhance the improvement of depressive and anxious symptoms (102). Some scholars propose that continuous theta-burst stimulation (cTBS) is effective for GAD. Comparisons with rTMS indicate that while cTBS typically produces inhibitory effects, it demonstrates superior symptom response and remission at one-month follow-up, potentially linked to enhanced alpha-band frequency, thereby improving psychological symptoms in GAD patients (103). However, these findings have not yet be adequately replicated in rigorously designed randomized controlled trials.

From a mechanistic perspective, rTMS may influence the neurobiological underpinnings of GAD through multiple pathways: modulating functional connectivity between the prefrontal cortex and limbic systems; affecting hypothalamic-pituitary-adrenal axis activity to reduce stress responses; regulating the balance between gamma-aminobutyric acid and glutamatergic neurotransmission; and alleviating neuroinflammation by decreasing pro-inflammatory cytokine levels. Nevertheless, most of these mechanisms currently remain largely hypothetical and require further direct evidence to substantiate them. Future research should systematically validate the precise mechanisms and clinical value of rTMS in treating GAD through large-scale, multicenter randomized controlled trials, incorporating standardized neurochemical assessments and neuroimaging monitoring.

## Challenges and prospects

As a non-invasive neurostimulation technique, rTMS’s core advantage lies in its ability to modulate the excitability of specific brain regions and networks in a relatively safe and controllable manner. Clinical evidence indicates that rTMS has demonstrated clear efficacy in treating symptoms such as Depressive Disorders, obsessive-compulsive disorder, and auditory hallucinations in schizophrenia, particularly offering new therapeutic options for patients with suboptimal response to medication. Nevertheless, the technology retains several critical limitations: although the efficacy of rTMS has been partially substantiated, its precise neurobiological mechanisms of action remain incompletely elucidated. Existing research predominantly relies on simplistic ‘inhibition’ or ‘excitation’ models, whereas rTMS may exert effects through multiple pathways, including modulation of neuroplasticity, brain network connectivity, neurotransmitter balance, and glial cell function. These mechanisms vary across different disorders and individuals, and current data remain insufficient in elucidating causal pathways. The definition of ‘optimal’ stimulation parameters remains a central controversy within the field. There is a lack of unified standards based on high-level evidence regarding the selection of high-frequency versus low-frequency stimulation, the relative merits of unilateral versus bilateral stimulation, and the appropriate treatment duration and maintenance protocols. This uncertainty directly contributes to significant heterogeneity in both clinical practice and scientific research, necessitating particular caution when interpreting meta-analysis results across studies. Most randomized controlled trials feature short follow-up periods, making it difficult to accurately reflect the long-term efficacy stability and potential long-term safety concerns of rTMS. For instance, reports indicate high relapse rates among OCD patients following acute treatment cessation, posing challenges for long-term therapeutic strategies. Existing data on long-term safety predominantly originate from open-label studies or case series, whose evidence level is limited. Establishing a systematic long-term follow-up monitoring system is urgently required.

To address these challenges, future research must advance simultaneously on two fronts: technological innovation and methodological refinement. Technologically, priority should be given to developing real-time closed-loop control systems. This could involve exploring the construction of real-time fMRI-EEG closed-loop feedback systems, which synchronously acquire blood oxygenation signals and electrophysiological activity to enable dynamic monitoring of neuromodulation effects and adaptive parameter adjustment. Coil design must overcome existing limitations by developing novel coil arrays capable of precisely targeting deep brain regions (e.g., nucleus accumbens, subgenual anterior cingulate cortex), combined with computational electromagnetics modelling to optimize field distribution characteristics. These engineering breakthroughs will significantly enhance rTMS’s capacity to intervene in critical neural circuits.

Methodologically, personalized neuromodulation should be grounded in multi-omics data integration. Connectome features should identify individual-specific network abnormalities, while genetic markers (e.g., BDNF Val66Met, COMT Val158Met) should predict treatment response. This approach will establish quantifiable standards for target localization and parameter setting. Implementation may be structured in three phases: initially pilot studies for connectome-based target identification, followed by multicenter randomized controlled trials to validate efficacy, culminating in a real-world research framework incorporating long-term follow-up.

Regarding long-term efficacy and safety, existing data indicate substantial inter-individual variability in rTMS efficacy maintenance. Obsessive-compulsive disorder patients exhibit high relapse rates within six months post-acute treatment cessation, underscoring the need for standardized maintenance protocols. It is recommended to develop individualized maintenance regimens based on acute-phase response, including monthly consolidation stimulation (1–2 sessions) or intermittent booster courses. Safety monitoring should follow standardized protocols integrating neurophysiological indicators, behavioral scales (YMRS, HARS), and patient-reported outcomes. An adverse event early warning system should be established, alongside optimized stimulation parameters (e.g., employing intermittent theta burst stimulation instead of conventional high-frequency stimulation), enhanced real-time monitoring during treatment, and a comprehensive long-term follow-up system.

Through sustained technological innovation and rigorous clinical research in the aforementioned key areas, rTMS holds promise to overcoming current limitations in the future. It may evolve from a promising technology into a precise, effective, and reliable component within comprehensive treatment system for psychiatric disorders.

## References

[1] Tse JSY Haslam N . What is a mental disorder? Evaluating the lay concept of mental ill health in the United States. BMC Psychiatry. (2023) 23:224. doi: 10.1186/s12888-023-04680-5 37013532 PMC10069095

[2] Prince M Patel V Saxena S Maj M Maselko J Phillips MR . No health without mental health. Lancet. (2007) 370:859–77. doi: 10.1016/s0140-6736(07)61238-0 17804063

[3] Patel V Chisholm D Parikh R Charlson FJ Degenhardt L Dua T . Addressing the burden of mental, neurological, and substance use disorders: key messages from Disease Control Priorities, 3rd edition. Lancet. (2016) 387:1672–85. doi: 10.1016/s0140-6736(15)00390-6 26454360

[4] Deng Y Sun S Wu S Chen K Liu Y Wei W . Burden and trends of mental disorders in China from 1990 to 2019: findings from the Global Burden of Disease Study 2019. Soc Psychiatry Psychiatr Epidemiol. (2024) 59:1563–76. doi: 10.1007/s00127-023-02594-x 38087123

[5] Brewster PR Mohammad Ishraq Bari S Walker GM Werfel TA . Current and future directions of drug delivery for the treatment of mental illnesses. Adv Drug Delivery Rev. (2023) 197:114824. doi: 10.1016/j.addr.2023.114824 37068660 PMC11479664

[6] Ferrarelli F Phillips ML . Examining and modulating neural circuits in psychiatric disorders with transcranial magnetic stimulation and electroencephalography: present practices and future developments. Am J Psychiatry. (2021) 178:400–13. doi: 10.1176/appi.ajp.2020.20071050 33653120 PMC8119323

[7] Clapp WC Hamm JP Kirk IJ Teyler TJ . Translating long-term potentiation from animals to humans: a novel method for noninvasive assessment of cortical plasticity. Biol Psychiatry. (2012) 71:496–502. doi: 10.1016/j.biopsych.2011.08.021 21974785 PMC3253317

[8] Klomjai W Katz R Lackmy-Vallée A . Basic principles of transcranial magnetic stimulation (TMS) and repetitive TMS (rTMS). Ann Phys Rehabil Med. (2015) 58:208–13. doi: 10.1016/j.rehab.2015.05.005 26319963

[9] Berardelli A Inghilleri M Rothwell JC Romeo S Currà A Gilio F . Facilitation of muscle evoked responses after repetitive cortical stimulation in man. Exp Brain Res. (1998) 122:79–84. doi: 10.1007/s002210050493 9772114

[10] Iyer MB Schleper N Wassermann EM . Priming stimulation enhances the depressant effect of low-frequency repetitive transcranial magnetic stimulation. J Neurosci. (2003) 23:10867–72. doi: 10.1523/jneurosci.23-34-10867.2003 14645480 PMC6740990

[11] Janicak PG O'Reardon JP Sampson SM Husain MM Lisanby SH Rado JT . Transcranial magnetic stimulation in the treatment of major depressive disorders: a comprehensive summary of safety experience from acute exposure, extended exposure, and during reintroduction treatment. J Clin Psychiatry. (2008) 69:222–32. doi: 10.4088/jcp.v69n0315 18232722

[12] Mutz J Edgcumbe DR Brunoni AR Fu CHY . Efficacy and acceptability of non-invasive brain stimulation for the treatment of adult unipolar and bipolar depression: a systematic review and meta-analysis of randomised sham-controlled trials. Neurosci Biobehav Rev. (2018) 92:291–303. doi: 10.1016/j.neubiorev.2018.05.015 29763711

[13] Salomons TV Dunlop K Kennedy SH Flint A Geraci J Giacobbe P . Resting-state cortico-thalamic-striatal connectivity predicts response to dorsomedial prefrontal rTMS in major depressive disorders. Neuropsychopharmacology. (2014) 39:488–98. doi: 10.1038/npp.2013.222 24150516 PMC3870791

[14] Blumberger DM Vila-Rodriguez F Thorpe KE Feffer K Noda Y Giacobbe P . Effectiveness of theta burst versus high-frequency repetitive transcranial magnetic stimulation in patients with depression (THREE-D): a randomised non-inferiority trial. Lancet. (2018) 391:1683–92. doi: 10.1016/s0140-6736(18)30295-2 29726344

[15] Neuteboom D Zantvoord JB Goya-Maldonado R Wilkening J Dols A van Exel E . Accelerated intermittent theta burst stimulation in major depressive disorders: a systematic review. Psychiatry Res. (2023) 327:115429. doi: 10.1016/j.psychres.2023.115429 37625365

[16] Gromann PM Tracy DK Giampietro V Brammer MJ Krabbendam L Shergill SS . Examining frontotemporal connectivity and rTMS in healthy controls: implications for auditory hallucinations in schizophrenia. Neuropsychology. (2012) 26:127–32. doi: 10.1037/a0026603 22409340

[17] Hasan A Wobrock T Guse B Langguth B Landgrebe M Eichhammer P . Structural brain changes are associated with response of negative symptoms to prefrontal repetitive transcranial magnetic stimulation in patients with schizophrenia. Mol Psychiatry. (2017) 22:857–64. doi: 10.1038/mp.2016.161 27725655

[18] Limongi R Mackinley M Dempster K Khan AR Gati JS Palaniyappan L . Frontal-striatal connectivity and positive symptoms of schizophrenia: implications for the mechanistic basis of prefrontal rTMS. Eur Arch Psychiatry Clin Neurosci. (2021) 271:3–15. doi: 10.1007/s00406-020-01163-6 32683527 PMC7867561

[19] Del Casale A Kotzalidis GD Rapinesi C Serata D Ambrosi E Simonetti A . Functional neuroimaging in obsessive-compulsive disorder. Neuropsychobiology. (2011) 64:61–85. doi: 10.1159/000325223 21701225

[20] Fineberg NA Chamberlain SR Hollander E Boulougouris V Robbins TW . Translational approaches to obsessive-compulsive disorder: from animal models to clinical treatment. Br J Pharmacol. (2011) 164:1044–61. doi: 10.1111/j.1476-5381.2011.01422.x 21486280 PMC3229751

[21] Milad MR Rauch SL . Obsessive-compulsive disorder: beyond segregated cortico-striatal pathways. Trends Cognit Sci. (2012) 16:43–51. doi: 10.1016/j.tics.2011.11.003 22138231 PMC4955838

[22] Haber SN . The primate basal ganglia: parallel and integrative networks. J Chem Neuroanat. (2003) 26:317–30. doi: 10.1016/j.jchemneu.2003.10.003 14729134

[23] Ochsner KN Silvers JA Buhle JT . Functional imaging studies of emotion regulation: a synthetic review and evolving model of the cognitive control of emotion. Ann N Y Acad Sci. (2012) 1251:E1–E24. doi: 10.1111/j.1749-6632.2012.06751.x 23025352 PMC4133790

[24] Diefenbach GJ Bragdon LB Zertuche L Hyatt CJ Hallion LS Tolin DF . Repetitive transcranial magnetic stimulation for generalised anxiety disorder: a pilot randomised, double-blind, sham-controlled trial. Br J Psychiatry. (2016) 209:222–8. doi: 10.1192/bjp.bp.115.168203 27198484

[25] Andreescu C Sheu LK Tudorascu D Gross JJ Walker S Banihashemi L . Emotion reactivity and regulation in late-life generalized anxiety disorder: functional connectivity at baseline and post-treatment. Am J Geriatr Psychiatry. (2015) 23:200–14. doi: 10.1016/j.jagp.2014.05.003 24996397 PMC4234701

[26] Monroe SM Harkness KL . Major depression and its recurrences: life course matters. Annu Rev Clin Psychol. (2022) 18:329–57. doi: 10.1146/annurev-clinpsy-072220-021440 35216520

[27] Dobrek L Głowacka K . Depression and its phytopharmacotherapy-a narrative review. Int J Mol Sci. (2023) 24. doi: 10.3390/ijms24054772 36902200 PMC10003400

[28] McGirr A Van den Eynde F Tovar-Perdomo S Fleck MP Berlim MT . Effectiveness and acceptability of accelerated repetitive transcranial magnetic stimulation (rTMS) for treatment-resistant major depressive disorders: an open label trial. J Affect Disord. (2015) 173:216–20. doi: 10.1016/j.jad.2014.10.068 25462419

[29] Berlim MT Van den Eynde F Daskalakis ZJ . High-frequency repetitive transcranial magnetic stimulation accelerates and enhances the clinical response to antidepressants in major depression: a meta-analysis of randomized, double-blind, and sham-controlled trials. J Clin Psychiatry. (2013) 74:e122–9. doi: 10.4088/jcp.12r07996 23473357

[30] De Raedt R Vanderhasselt MA Baeken C . Neurostimulation as an intervention for treatment resistant depression: from research on mechanisms towards targeted neurocognitive strategies. Clin Psychol Rev. (2015) 41:61–9. doi: 10.1016/j.cpr.2014.10.006 25468571

[31] Eche J Mondino M Haesebaert F Saoud M Poulet E Brunelin J . Low- vs high-frequency repetitive transcranial magnetic stimulation as an add-on treatment for refractory depression. Front Psychiatry. (2012) 3:13. doi: 10.3389/fpsyt.2012.00013 22408627 PMC3296064

[32] Berlim MT Van den Eynde F Daskalakis ZJ . Clinically meaningful efficacy and acceptability of low-frequency repetitive transcranial magnetic stimulation (rTMS) for treating primary major depression: a meta-analysis of randomized, double-blind and sham-controlled trials. Neuropsychopharmacology. (2013) 38:543–51. doi: 10.1038/npp.2012.237 23249815 PMC3572468

[33] Hsu TW Yeh TC Kao YC Thompson T Brunoni AR Carvalho AF . The dose-effect relationship of six stimulation parameters with rTMS over left DLPFC on treatment-resistant depression: a systematic review and meta-analysis. Neurosci Biobehav Rev. (2024) 162:105704. doi: 10.1016/j.neubiorev.2024.105704 38723735

[34] Berlim MT Van den Eynde F Daskalakis ZJ . Efficacy and acceptability of high frequency repetitive transcranial magnetic stimulation (rTMS) versus electroconvulsive therapy (ECT) for major depression: a systematic review and meta-analysis of randomized trials. Depress Anxiety. (2013) 30:614–23. doi: 10.1002/da.22060 23349112

[35] Baeken C Brem AK Arns M Brunoni AR Filipčić I Ganho-Ávila A . Repetitive transcranial magnetic stimulation treatment for depressive disorders: current knowledge and future directions. Curr Opin Psychiatry. (2019) 32:409–15. doi: 10.1097/yco.0000000000000533 31145145 PMC6688778

[36] Maneeton B Maneeton N Woottiluk P Likhitsathian S . Repetitive transcranial magnetic stimulation combined with antidepressants for the first episode of major depressive disorders. Curr Neuropharmacol. (2020) 18:852–60. doi: 10.2174/1570159x18666200221113134 32091338 PMC7569318

[37] Liu B Zhang Y Zhang L Li L . Repetitive transcranial magnetic stimulation as an augmentative strategy for treatment-resistant depression, a meta-analysis of randomized, double-blind and sham-controlled study. BMC Psychiatry. (2014) 14:342. doi: 10.1186/s12888-014-0342-4 25433539 PMC4264336

[38] Dębowska W Więdłocha M Dębowska M Kownacka Z Marcinowicz P Szulc A . Transcranial magnetic stimulation and ketamine: implications for combined treatment in depression. Front Neurosci. (2023) 17:1267647. doi: 10.3389/fnins.2023.1267647 37954877 PMC10637948

[39] Donse L Padberg F Sack AT Rush AJ Arns M . Simultaneous rTMS and psychotherapy in major depressive disorders: clinical outcomes and predictors from a large naturalistic study. Brain Stimul. (2018) 11:337–45. doi: 10.1016/j.brs.2017.11.004 29174304

[40] Wu W Zhang Y Jiang J Lucas MV Fonzo GA Rolle CE . An electroencephalographic signature predicts antidepressant response in major depression. Nat Biotechnol. (2020) 38:439–47. doi: 10.1038/s41587-019-0397-3 32042166 PMC7145761

[41] Tondo L Vázquez GH Baldessarini RJ . Depression and mania in bipolar disorder. Curr Neuropharmacol. (2017) 15:353–8. doi: 10.2174/1570159x14666160606210811 28503106 PMC5405618

[42] Bonnín CDM Reinares M Martínez-Arán A Jiménez E Sánchez-Moreno J Solé B . Improving functioning, quality of life, and well-being in patients with bipolar disorder. Int J Neuropsychopharmacol. (2019) 22:467–77. doi: 10.1093/ijnp/pyz018 PMC6672628 31093646

[43] George MS Nahas Z Molloy M Speer AM Oliver NC Li XB . A controlled trial of daily left prefrontal cortex TMS for treating depression. Biol Psychiatry. (2000) 48:962–70. doi: 10.1016/s0006-3223(00)01048-9 11082469

[44] Sheline YI Makhoul W Batzdorf AS Nitchie FJ Lynch KG Cash R . Accelerated intermittent theta-burst stimulation and treatment-refractory bipolar depression: a randomized clinical trial. JAMA Psychiatry. (2024) 81:936–41. doi: 10.1001/jamapsychiatry.2024.1787 38985492 PMC11238064

[45] Torres IJ Ge R McGirr A Vila-Rodriguez F Ahn S Basivireddy J . Effects of intermittent theta-burst transcranial magnetic stimulation on cognition and hippocampal volumes in bipolar depression. Dialogues Clin Neurosci. (2023) 25:24–32. doi: 10.1080/19585969.2023.2186189 36924413 PMC10026761

[46] Baeken C Wu G Sackeim HA . Accelerated iTBS treatment applied to the left DLPFC in depressed patients results in a rapid volume increase in the left hippocampal dentate gyrus, not driven by brain perfusion. Brain Stimul. (2020) 13:1211–7. doi: 10.1016/j.brs.2020.05.015 32512184

[47] Dell'Osso B Mundo E D'Urso N Pozzoli S Buoli M Ciabatti M . Augmentative repetitive navigated transcranial magnetic stimulation (rTMS) in drug-resistant bipolar depression. Bipolar Disord. (2009) 11:76–81. doi: 10.1111/j.1399-5618.2008.00651.x 19133969

[48] Dell'Osso B Oldani L Camuri G Dobrea C Cremaschi L Benatti B . Augmentative repetitive transcranial magnetic stimulation (rTMS) in the acute treatment of poor responder depressed patients: a comparison study between high and low frequency stimulation. Eur Psychiatry. (2015) 30:271–6. doi: 10.1016/j.eurpsy.2014.12.001 25572482

[49] Harel EV Zangen A Roth Y Reti I Braw Y Levkovitz Y . H-coil repetitive transcranial magnetic stimulation for the treatment of bipolar depression: an add-on, safety and feasibility study. World J Biol Psychiatry. (2011) 12:119–26. doi: 10.3109/15622975.2010.510893 20854181

[50] Dell'Osso B D'Urso N Castellano F Ciabatti M Altamura AC . Long-term efficacy after acute augmentative repetitive transcranial magnetic stimulation in bipolar depression: a 1-year follow-up study. J ECT. (2011) 27:141–4. doi: 10.1097/YCT.0b013e3181f66601 20966770

[51] Grisaru N Chudakov B Yaroslavsky Y Belmaker RH . Transcranial magnetic stimulation in mania: a controlled study. Am J Psychiatry. (1998) 155:1608–10. doi: 10.1176/ajp.155.11.1608 9812128

[52] Praharaj SK Ram D Arora M . Efficacy of high frequency (rapid) suprathreshold repetitive transcranial magnetic stimulation of right prefrontal cortex in bipolar mania: a randomized sham controlled study. J Affect Disord. (2009) 117:146–50. doi: 10.1016/j.jad.2008.12.020 19178948

[53] Pathak V Sinha VK Praharaj SK . Efficacy of adjunctive high frequency repetitive transcranial magnetic stimulation of right prefrontal cortex in adolescent mania: a randomized sham-controlled study. Clin Psychopharmacol Neurosci. (2015) 13:245–9. doi: 10.9758/cpn.2015.13.3.245 26598581 PMC4662165

[54] Kaptsan A Yaroslavsky Y Applebaum J Belmaker RH Grisaru N . Right prefrontal TMS versus sham treatment of mania: a controlled study. Bipolar Disord. (2003) 5:36–9. doi: 10.1034/j.1399-5618.2003.00003.x 12656936

[55] Hu SH Lai JB Xu DR Qi HL Peterson BS Bao AM . Efficacy of repetitive transcranial magnetic stimulation with quetiapine in treating bipolar II depression: a randomized, double-blinded, control study. Sci Rep. (2016) 6:30537. doi: 10.1038/srep30537 27460201 PMC4962310

[56] Gold AK Ornelas AC Cirillo P Caldieraro MA Nardi AE Nierenberg AA . Clinical applications of transcranial magnetic stimulation in bipolar disorder. Brain Behav. (2019) 9:e01419. doi: 10.1002/brb3.1419 31566935 PMC6790310

[57] Tandon R Gaebel W Barch DM Bustillo J Gur RE Heckers S . Definition and description of schizophrenia in the DSM-5. Schizophr Res. (2013) 150:3–10. doi: 10.1016/j.schres.2013.05.028 23800613

[58] Dougall N Maayan N Soares-Weiser K McDermott LM McIntosh A . Transcranial magnetic stimulation for schizophrenia. Schizophr Bull. (2015) 41:1220–2. doi: 10.1002/14651858.cd006081 26392626 PMC4601720

[59] Bowen EFW Burgess JL Granger R Kleinman JE Rhodes CH . DLPFC transcriptome defines two molecular subtypes of schizophrenia. Transl Psychiatry. (2019) 9:147. doi: 10.1101/116699 31073119 PMC6509343

[60] Mehta UM Thirthalli J Subbakrishna DK Gangadhar BN Eack SM Keshavan MS . Social and neuro-cognition as distinct cognitive factors in schizophrenia: a systematic review. Schizophr Res. (2013) 148:3–11. doi: 10.1016/j.schres.2013.05.009 23732017

[61] Mehta UM Thirthalli J Naveen Kumar C Keshav Kumar J Keshavan MS Gangadhar BN . Schizophrenia patients experience substantial social cognition deficits across multiple domains in remission. Asian J Psychiatr. (2013) 6:324–9. doi: 10.1016/j.ajp.2013.02.001 23810141

[62] Fett AK Viechtbauer W Dominguez MD Penn DL van Os J Krabbendam L . The relationship between neurocognition and social cognition with functional outcomes in schizophrenia: a meta-analysis. Neurosci Biobehav Rev. (2011) 35:573–88. doi: 10.1016/j.neubiorev.2010.07.001 20620163

[63] Mao J Fan K Zhang Y Wen N Fang X Ye X . 10 Hz repetitive transcranial magnetic stimulation (rTMS) may improve cognitive function: an exploratory study of schizophrenia patients with auditory hallucinations. Heliyon. (2023) 9:e19912. doi: 10.1016/j.heliyon.2023.e19912 37809845 PMC10559318

[64] Barr MS Farzan F Rajji TK Voineskos AN Blumberger DM Arenovich T . Can repetitive magnetic stimulation improve cognition in schizophrenia? Pilot data from a randomized controlled trial. Biol Psychiatry. (2013) 73:510–7. doi: 10.1016/j.biopsych.2012.08.020 23039931

[65] Francis MM Hummer TA Vohs JL Yung MG Visco AC Mehdiyoun NF . Cognitive effects of bilateral high frequency repetitive transcranial magnetic stimulation in early phase psychosis: a pilot study. Brain Imaging Behav. (2019) 13:852–61. doi: 10.1007/s11682-018-9902-4 29855992

[66] Hasan A Guse B Cordes J Wölwer W Winterer G Gaebel W . Cognitive effects of high-frequency rTMS in schizophrenia patients with predominant negative symptoms: results from a multicenter randomized sham-controlled trial. Schizophr Bull. (2016) 42:608–18. doi: 10.1093/schbul/sbv142 26433217 PMC4838079

[67] Wölwer W Lowe A Brinkmeyer J Streit M Habakuck M Agelink MW . Repetitive transcranial magnetic stimulation (rTMS) improves facial affect recognition in schizophrenia. Brain Stimul. (2014) 7:559–63. doi: 10.1159/000101033 24857264

[68] Llorca PM Pereira B Jardri R Chereau-Boudet I Brousse G Misdrahi D . Hallucinations in schizophrenia and Parkinson's disease: an analysis of sensory modalities involved and the repercussion on patients. Sci Rep. (2016) 6:38152. doi: 10.1038/srep38152 27905557 PMC5131286

[69] Shergill SS Brammer MJ Williams SC Murray RM McGuire PK . Mapping auditory hallucinations in schizophrenia using functional magnetic resonance imaging. Arch Gen Psychiatry. (2000) 57:1033–8. doi: 10.1001/archpsyc.57.11.1033 11074868

[70] Csukly G Orbán-Szigeti B Suri K Zsigmond R Hermán L Simon V . Theta-burst rTMS in schizophrenia to ameliorate negative and cognitive symptoms: study protocol for a double-blind, sham-controlled, randomized clinical trial. Trials. (2024) 25:269. doi: 10.1192/j.eurpsy.2024.455 38632647 PMC11025264

[71] Horacek J Brunovsky M Novak T Skrdlantova L Klirova M Bubenikova-Valesova V . Effect of low-frequency rTMS on electromagnetic tomography (LORETA) and regional brain metabolism (PET) in schizophrenia patients with auditory hallucinations. Neuropsychobiology. (2007) 55:132–42. doi: 10.1159/000106055 17641545

[72] Sommer IE de Weijer AD Daalman K Neggers SF Somers M Kahn RS . Can fMRI-guidance improve the efficacy of rTMS treatment for auditory verbal hallucinations? Schizophr Res. (2007) 93:406–8. doi: 10.1016/j.schres.2007.03.020 17478084

[73] Kamp D Brinkmeyer J Agelink MW Habakuck M Mobascher A Wölwer W . High frequency repetitive transcranial magnetic stimulation (rTMS) reduces EEG-hypofrontality in patients with schizophrenia. Psychiatry Res. (2016) 236:199–201. doi: 10.1016/j.psychres.2016.01.007 26778629

[74] Jin Y Potkin SG Kemp AS Huerta ST Alva G Thai TM . Therapeutic effects of individualized alpha frequency transcranial magnetic stimulation (alphaTMS) on the negative symptoms of schizophrenia. Schizophr Bull. (2006) 32:556–61. doi: 10.1093/schbul/sbj020 16254067 PMC2632240

[75] Klimesch W Sauseng P Gerloff C . Enhancing cognitive performance with repetitive transcranial magnetic stimulation at human individual alpha frequency. Eur J Neurosci. (2003) 17:1129–33. doi: 10.1046/j.1460-9568.2003.02517.x 12653991

[76] Aleman A Enriquez-Geppert S Knegtering H Dlabac-de Lange JJ . Moderate effects of noninvasive brain stimulation of the frontal cortex for improving negative symptoms in schizophrenia: meta-analysis of controlled trials. Neurosci Biobehav Rev. (2018) 89:111–8. doi: 10.1016/j.neubiorev.2018.02.009 29471017

[77] Kennedy NI Lee WH Frangou S . Efficacy of non-invasive brain stimulation on the symptom dimensions of schizophrenia: a meta-analysis of randomized controlled trials. Eur Psychiatry. (2018) 49:69–77. doi: 10.1016/j.eurpsy.2017.12.025 29413808

[78] Hoffman RE Hampson M Wu K Anderson AW Gore JC Buchanan RJ . Probing the pathophysiology of auditory/verbal hallucinations by combining functional magnetic resonance imaging and transcranial magnetic stimulation. Cereb Cortex. (2007) 17:2733–43. doi: 10.1093/cercor/bhl183 17298962 PMC2634833

[79] Schönfeldt-Lecuona C Grön G Walter H Büchler N Wunderlich A Spitzer M . Stereotaxic rTMS for the treatment of auditory hallucinations in schizophrenia. Neuroreport. (2004) 15:1669–73. doi: 10.1097/01.wnr.0000126504.89983.ec 15232304

[80] Francis MM Hummer TA Vohs JL Yung MG Liffick E Mehdiyoun NF . Functional neuroanatomical correlates of episodic memory impairment in early phase psychosis. Brain Imaging Behav. (2016) 10:1–11. doi: 10.1007/s11682-015-9357-9 25749917 PMC4561590

[81] Rapinesi C Kotzalidis GD Ferracuti S Sani G Girardi P Del Casale A . Brain stimulation in obsessive-compulsive disorder (OCD): a systematic review. Curr Neuropharmacol. (2019) 17:787–807. doi: 10.2174/1570159x17666190409142555 30963971 PMC7059162

[82] Stein DJ Koen N Fineberg N Fontenelle LF Matsunaga H Osser D . A 2012 evidence-based algorithm for the pharmacotherapy for obsessive-compulsive disorder. Curr Psychiatry Rep. (2012) 14:211–9. doi: 10.1007/s11920-012-0268-9 22527872

[83] Pallanti S Quercioli L . Treatment-refractory obsessive-compulsive disorder: methodological issues, operational definitions and therapeutic lines. Prog Neuro-Psychopharmacol Biol Psychiatry. (2006) 30:400–12. doi: 10.1016/j.pnpbp.2005.11.028 16503369

[84] Mantovani A Simpson HB Fallon BA Rossi S Lisanby SH . Randomized sham-controlled trial of repetitive transcranial magnetic stimulation in treatment-resistant obsessive-compulsive disorder. Int J Neuropsychopharmacol. (2010) 13:217–27. doi: 10.1017/s1461145709990435 19691873

[85] Fitzsimmons S van der Werf YD van Campen AD Arns M Sack AT Hoogendoorn AW . Repetitive transcranial magnetic stimulation for obsessive-compulsive disorder: a systematic review and pairwise/network meta-analysis. J Affect Disord. (2022) 302:302–12. doi: 10.1016/j.jad.2022.01.048 35041869

[86] Carmi L Tendler A Bystritsky A Hollander E Blumberger DM Daskalakis J . Efficacy and safety of deep transcranial magnetic stimulation for obsessive-compulsive disorder: a prospective multicenter randomized double-blind placebo-controlled trial. Am J Psychiatry. (2019) 176:931–8. doi: 10.1176/appi.ajp.2019.18101180 31109199

[87] Suhas S Malo PK Kumar V Issac TG Chithra NK Bhaskarapillai B . Treatment strategies for serotonin reuptake inhibitor-resistant obsessive-compulsive disorder: a network meta-analysis of randomised controlled trials. World J Biol Psychiatry. (2023) 24:162–77. doi: 10.1080/15622975.2022.2082525 35615998

[88] Harika-Germaneau G Rachid F Chatard A Lafay-Chebassier C Solinas M Thirioux B . Continuous theta burst stimulation over the supplementary motor area in refractory obsessive-compulsive disorder treatment: a randomized sham-controlled trial. Brain Stimul. (2019) 12:1565–71. doi: 10.1016/j.brs.2019.07.019 31383594

[89] Liang K Li H Bu X Li X Cao L Liu J . Efficacy and tolerability of repetitive transcranial magnetic stimulation for the treatment of obsessive-compulsive disorder in adults: a systematic review and network meta-analysis. Transl Psychiatry. (2021) 11:332. doi: 10.1038/s41398-021-01453-0 34050130 PMC8163761

[90] Steuber ER McGuire JF . A meta-analysis of transcranial magnetic stimulation in obsessive-compulsive disorder. Biol Psychiatry Cognit Neurosci Neuroimaging. (2023) 8:1145–55. doi: 10.1016/j.bpsc.2023.06.003 37343662

[91] Bandelow B Michaelis S . Epidemiology of anxiety disorders in the 21st century. Dialogues Clin Neurosci. (2015) 17:327–35. doi: 10.31887/dcns.2015.17.3/bbandelow 26487813 PMC4610617

[92] Strawn JR Lu L Peris TS Levine A Walkup JT . Research review: pediatric anxiety disorders - what have we learnt in the last 10 years? J Child Psychol Psychiatry. (2021) 62:114–39. doi: 10.1111/jcpp.13262 32500537 PMC7718323

[93] Tyrer P Baldwin D . Generalised anxiety disorder. Lancet. (2006) 368:2156–66. doi: 10.1016/s0140-6736(06)69865-6 17174708

[94] Ansara ED . Management of treatment-resistant generalized anxiety disorder. Ment Health Clin. (2020) 10:326–34. doi: 10.9740/mhc.2020.11.326 33224690 PMC7653736

[95] Parikh TK Strawn JR Walkup JT Croarkin PE . Repetitive transcranial magnetic stimulation for generalized anxiety disorder: a systematic literature review and meta-analysis. Int J Neuropsychopharmacol. (2022) 25:144–6. doi: 10.1093/ijnp/pyab077 34791241 PMC8832221

[96] Guo B Zhang M Hao W Wang Y Zhang T Liu C . Neuroinflammation mechanisms of neuromodulation therapies for anxiety and depression. Transl Psychiatry. (2023) 13:5. doi: 10.1038/s41398-022-02297-y 36624089 PMC9829236

[97] Fitzgerald PB Hoy KE Reynolds J Singh A Gunewardene R Slack C . A pragmatic randomized controlled trial exploring the relationship between pulse number and response to repetitive transcranial magnetic stimulation treatment in depression. Brain Stimul. (2020) 13:145–52. doi: 10.1016/j.brs.2019.09.001 31521543

[98] Weissman CR Blumberger DM Brown PE Isserles M Rajji TK Downar J . Bilateral repetitive transcranial magnetic stimulation decreases suicidal ideation in depression. J Clin Psychiatry. (2018) 79. doi: 10.4088/JCP.17m11692 29701939

[99] Dilkov D Hawken ER Kaludiev E Milev R . Repetitive transcranial magnetic stimulation of the right dorsal lateral prefrontal cortex in the treatment of generalized anxiety disorder: a randomized, double-blind sham controlled clinical trial. Prog Neuro-Psychopharmacol Biol Psychiatry. (2017) 78:61–5. doi: 10.1016/j.pnpbp.2017.05.018 28533148

[100] Lu R Zhang C Liu Y Wang L Chen X Zhou X . The effect of bilateral low-frequency rTMS over dorsolateral prefrontal cortex on serum brain-derived neurotropic factor and serotonin in patients with generalized anxiety disorder. Neurosci Lett. (2018) 684:67–71. doi: 10.1016/j.neulet.2018.07.008 30008380

[101] White D Tavakoli S . Repetitive transcranial magnetic stimulation for treatment of major depressive disorders with comorbid generalized anxiety disorder. Ann Clin Psychiatry. (2015) 27:192–6. doi: 10.1177/104012371502700305 26247218

[102] Wang L Zhou QH Wang K Wang HC Hu SM Yang YX . Frontoparietal paired associative stimulation versus single-site stimulation for generalized anxiety disorder: a pilot rTMS study. J Psychiatry Neurosci. (2022) 47:E153–e161. doi: 10.1503/jpn.210201 35477683 PMC9259432

[103] Li X Zhang C Tan J Ding L Wang C Wang M . Clinical effects of continuous theta burst stimulation for generalized anxiety disorder and a mechanism involving α oscillations: a randomized controlled trial. J Psychiatry Neurosci. (2022) 47:E123–e133. doi: 10.1503/jpn.210134 35361700 PMC8979658

